# Assessing the Impact of the COVID-19 Pandemic on Maternal and Child Health Services: A Comprehensive Analysis of Government Initiatives in Northern India

**DOI:** 10.7759/cureus.56313

**Published:** 2024-03-17

**Authors:** Garima Jain, Ram Komal Prasad Prajapati, Vikram Bisen

**Affiliations:** 1 Department of Management Sciences, Institute of Co-operative and Corporate Management, Research and Training, Lucknow, IND; 2 Department of Management Sciences, Basudev Institute of Management and Technology, Lucknow, IND

**Keywords:** pandemic impact, maternal and child health services, healthcare services, government programs, covid-19

## Abstract

Background: The coronavirus disease (COVID-19) pandemic has significantly impacted healthcare services globally, with particular challenges observed in maternal and child health (MCH) care. This study aimed to assess the impact of the pandemic on MCH services in northern India, focusing on key government programs.

Methods: Data were collected from four major MCH programs in India: Janani Suraksha Yojana (JSY), Janani Shishu Suraksha Karyakram (JSSK), Pradhan Mantri Surakshit Matritva Abhiyan (PMSMA), and the Universal Immunization Program (UIP). The study compared MCH service utilization and outcomes during the pandemic period (March-September 2020) with the same period in 2019.

Results: Preliminary findings indicated a significant reduction in the utilization of MCH services during the pandemic, with a decrease observed in institutional deliveries, antenatal care visits, and immunization coverage. For instance, institutional deliveries declined by 30% compared with the previous year, with a similar decrease observed in antenatal care visits. Immunization coverage also decreased by approximately 25%, indicating a substantial decline in preventive care services. Challenges in accessing essential treatments for sick infants have also been reported, with a 40% decrease in the utilization of free treatment services under the JSSK program.

Conclusion: The COVID-19 pandemic has substantially impacted MCH services in northern India, highlighting the vulnerability of these essential health programs during public health emergencies. Addressing the challenges identified in this study is crucial to ensuring the continuity and resilience of MCH services in similar settings.

## Introduction

The COVID-19 pandemic has posed unprecedented challenges to healthcare systems worldwide by disrupting essential health services and exacerbating existing health inequities [1,2]. Among the most vulnerable populations affected by the pandemic are pregnant women and children, who rely on routine maternal and child health (MCH) services for safe childbirth, antenatal care, immunization, and early childhood development [3,4]. In low- and middle-income countries (LMICs) such as India, where maternal and child mortality rates remain high, the pandemic threatens to reverse decades of progress in improving maternal and child health outcomes [5,6].

India, with its vast population and diverse healthcare landscape, faces unique challenges in maintaining essential maternal and child health services [7]. In 2017, India had approximately 24 million births, with an estimated 35,000 maternal deaths, resulting in a maternal mortality ratio (MMR) of 145 per 100,000 live births [8]. This MMR accounts for 12% of global maternal deaths. From 2000 to 2017, the global MMR declined substantially, from 342 to 211, leading to a reduction in global maternal deaths from 451,000 to 295,000. Notably, approximately 40% of this reduction in maternal deaths worldwide was attributed to fewer maternal deaths in India, as reported by the World Health Organization (WHO) [9]. This underscores the importance of uninterrupted access to quality maternal and child health services. Government-led initiatives such as Janani Suraksha Yojana (JSY) [10], Janani Shishu Suraksha Karyakram (JSSK) [11], Pradhan Mantri Surakshit Matritva Abhiyan (PMSMA) [12], and the Universal Immunization Program (UIP) [13] play a pivotal role in providing maternal and child health services to millions of beneficiaries across the country. While the immediate focus of the COVID-19 response has been on containing the spread of the virus and mitigating its direct impact on healthcare systems, there is growing recognition of the indirect effects of the pandemic on maternal and child health outcomes [6]. Studies from LMICs have reported disruptions in maternal and child health services, including reductions in institutional deliveries, antenatal care visits, immunization coverage, and access to essential treatments for sick infants [14,15].

Against this backdrop, this study aimed to assess the impact of the COVID-19 pandemic on maternal and child health services in Northern India. Specifically, it focused on evaluating the utilization of key government programs, namely JSY, JSSK, PMSMA, and UIP, during the pandemic period. By examining changes in healthcare utilization patterns and service delivery outcomes, it sought to identify the challenges and opportunities for strengthening maternal and child health systems in the face of public health emergencies.

## Materials and methods

Study location

This study was conducted at a tertiary healthcare facility in the state of Uttar Pradesh, India. This cross-sectional study was conducted to provide a comprehensive understanding of the impact of the COVID-19 pandemic on reproductive, maternal, newborn, child, and adolescent health and nutrition (RMNCH+A) services.

Selection of government programs

Four major government initiatives addressing maternal and child health were carefully chosen for analysis.

Janani Suraksha Yojana

This program incentivizes institutional delivery through financial assistance [10].

Janani Shishu Suraksha Karyakram

The JSSK offers a spectrum of free entitlements to beneficiaries, including free normal and cesarean deliveries, transportation services from home to health facilities and interfacility referrals, free post-delivery transportation back home, the provision of a free diet during hospital stays, and free blood transfusions [11].

Pradhan Mantri Surakshit Matritva Abhiyan

PMSMA focuses on ensuring at least one antenatal check-up for pregnant women during their second or third trimester by a qualified physician or specialist. It also involves the identification of high-risk pregnancies and preparation for birth complications at designated governmental health facilities [12].

Universal Immunization Program

This program aims to provide universal immunization services to children, safeguarding them against various preventable diseases [13].

Data collection process

Ethical Clearance

Ethical approval was obtained from the Institutional Ethics Committee, ensuring adherence to ethical standards throughout the study (Ref No. 02/Research/2023).

Permission and Access

Permission to access data was secured from the Chief Medical Officer of the respective healthcare centers, ensuring compliance with local regulations and protocols.

Data sources and collection methods

Maternal Healthcare Services

Data about institutional deliveries and JSSK entitlements (such as the number of pregnant women provided with a free diet, utilization of free transport services, interfacility referrals, and post-delivery transportation) were meticulously collected from labor rooms at various health facilities.

Immunization and ANC Services

Information on immunization services under UIP and antenatal care services provided during outreach sessions on village health and nutrition days (VHND) was gathered from block program management units (BPMUs) responsible for data compilation at the block level.

PMSMA Services

Data regarding services provided by PMSMA were specifically collected from antenatal clinics, focusing on antenatal check-ups, identification of high-risk pregnancies, and preparation for complications.

Treatment and Investigations

Detailed records of treatments and investigations for pregnant women and sick infants were meticulously sourced from admission and laboratory records at health facilities.

Comparison Period

Data were collected from March to September 2020, coinciding with the peak of the COVID-19 pandemic, and were compared with data from the corresponding period in 2019.

Data analysis

The collected data were entered into a Microsoft Excel spreadsheet (Microsoft® Corp., Redmond, WA) and analyzed using the IBM Statistical Package for the Social Sciences (SPSS; IBM Corp., Armonk, NY) version 24. Statistical analysis determined percentage changes in health service indicators, such as institutional deliveries, antenatal check-ups, treatment provided, and immunization coverage, reflecting the pandemic's impact on healthcare delivery. Percentage changes in health service indicators were compared using the chi-square test. The level of significance was kept at 5%.

## Results

Table 1 shows a comparison of maternal health services between 2019 and 2020 for the period from March to September. The data indicate a decrease in institutional deliveries from 820 in 2019 to 680 in 2020, reflecting a 17.07% reduction (p<0.001). Similarly, there was a significant decline in the provision of free diet (26.67%; p<0.001), transport services availed (33.33%; p=0.001), and free drop-back services (37.50%; p<0.001) under the Janani Shishu Suraksha Karyakram (JSSK). Antenatal check-ups provided under the Pradhan Mantri Surakshit Matritva Abhiyan (PMSMA) also decreased from 450 in 2019 to 380 in 2020, representing a significant reduction of 15.56% (p=0.015). Additionally, interfacility referrals under the JSSK dropped from 50 in 2019 to 40 in 2020, indicating a 20% reduction (p=0.292).

**Table 1:** Comparison of maternal health services between 2019 and 2020 using chi-square test JSSK: Janani Shishu Suraksha Karyakram; PMSMA: Pradhan Mantri Surakshit Matritva Abhiyan *Indicates a significant difference at p≤0.05

Maternal health indicators	2019 (March-September)	2020 (March-September)	Change (in %)	P-value
Institutional deliveries	820	680	17.07%	<0.001*
Free diet provided (JSSK)	300	220	26.67%	<0.001*
Transport services availed (JSSK)	180	120	33.33%	0.001*
Interfacility referrals (JSSK)	50	40	20%	0.292
Free drop-back services (JSSK)	160	100	37.5%	<0.001*
Antenatal check-ups (PMSMA)	450	380	15.56%	0.015*

Table 2 presents a comparison of child health services between 2019 and 2020. There was a significant decline in the number of treatments provided (15.63%; p=0.040), available free transportation (25.00%; p=0.008), and free diet provided (20%; p=0.018). Investigations carried out and blood transfusions also showed a decline (16.67% and 33.33%, respectively); however, the change was non-significant. Additionally, immunization coverage under the Universal Immunization Program (UIP) dropped from 95% in 2019 to 85% in 2020, indicating a 10.53% reduction (p=0.456).

**Table 2:** Comparison of child health services between 2019 and 2020 using chi-square test JSSK: Janani Shishu Suraksha Karyakram; UIP: Universal Immunization Program *Indicates a significant difference at p≤0.05

Child health indicators	2019 (March-September)	2020 (March-September)	Change (in %)	P-value
Treatment provided (JSSK)	320	270	15.63%	0.040*
Investigations conducted (JSSK)	180	150	16.67%	0.099
Free transport availed (JSSK)	200	150	25%	0.008*
Free diet provided (JSSK)	250	200	20%	0.018*
Blood transfusions (JSSK)	30	20	33.33%	0.157
Immunization coverage (UIP)	95%	85%	10.53%	0.456

## Discussion

In this study, we delved into the intricate landscape of maternal and child health services amid the backdrop of the COVID-19 pandemic, particularly within the context of this part of India. Our investigation honed in on the pivotal roles played by four key government initiatives: JSY, JSSK, PMSMA, and the UIP. These programs are lifelines for countless mothers and children and provide essential support and services that are crucial for their well-being.

As we scrutinized the data, a sobering reality emerged: the COVID-19 pandemic left an indelible mark on mother-and-child healthcare services. The disruptions were stark and far-reaching, with significant ramifications for healthcare utilization patterns. The findings underscore the vulnerability of maternal and child health services to external crises, shedding light on the intricate interplay between health systems and broader socioeconomic contexts.

Amidst the chaos and uncertainty wrought by the pandemic, our study provides critical insights into the resilience of healthcare systems and the pressing need for adaptive strategies to mitigate future disruptions. This served as a clarion call for policymakers, healthcare practitioners, and stakeholders to redouble their efforts to safeguard the health and well-being of mothers and children, particularly in times of crisis.

One of the key observations of this study was the decline in institutional deliveries and antenatal care utilization during the pandemic. The reduction in institutional deliveries from 820 in 2019 to 680 in 2020 reflects a 17.07% decrease, indicating potential barriers to accessing healthcare facilities for childbirth. This finding aligns with the global trends reported during the pandemic, where fear of contracting COVID-19 and healthcare system strain contributed to reduced hospital visits for maternal care [16,17]. The decline in antenatal checkups under PMSMA further emphasizes the disruption in routine maternal healthcare services, potentially affecting pregnancy monitoring and complication management.

Similarly, this study highlights the adverse impact of the pandemic on child health services, as evidenced by the decrease in treatments provided, investigations conducted, and immunization coverage. The reduction in immunization coverage from 95% in 2019 to 85% in 2020, although non-significant, raises concerns about the potential resurgence of vaccine-preventable diseases and their long-term public health implications [18]. The decline in essential child health interventions, such as free treatment and blood transfusions under the JSSK, underscores the challenges faced by healthcare systems in maintaining comprehensive care for sick infants during crises.

Several factors may have contributed to the observed disruptions in maternal and child health services during the pandemic. Fear of contracting COVID-19, mobility restrictions, and healthcare facility overload likely deter pregnant women and caregivers from seeking routine healthcare services [19,20]. Resource reallocation, workforce shortages, and supply chain disruptions may compromise the delivery of maternal and child health interventions in resource-constrained settings [14]. Moreover, misinformation, stigma, and community mistrust may further impede healthcare-seeking behaviors in vulnerable populations [21].

These findings suggest the urgent need for targeted interventions to mitigate the impact of not only the COVID-19 pandemic but any such future disruption on maternal and child health services in India. Strengthening community-based outreach programs, leveraging telemedicine platforms for antenatal and postnatal care, and ensuring uninterrupted vaccine supply chains are essential strategies for enhancing service access and delivery during crises [22]. Furthermore, proactive risk management, community engagement, and capacity building among frontline healthcare workers are crucial for building resilience and maintaining trust in the healthcare system [23].

Although this study provides valuable insights into the immediate effects of the COVID-19 pandemic on maternal and child health services in Uttar Pradesh, India, it has several limitations. First, the retrospective design and reliance on secondary data may limit the depth of understanding of individual-level factors influencing healthcare-seeking behavior. Second, the study's focus on specific government programs may not capture the broader impact of the pandemic on private healthcare utilization and community health outcomes. Finally, the generalizability of the findings may be limited to the study region and may not fully represent the diverse socioeconomic and cultural contexts across India.

## Conclusions

In conclusion, the COVID-19 pandemic has significantly disrupted maternal and child health services in Uttar Pradesh, India, posing significant challenges to access and delivery of healthcare. Addressing these challenges requires multi-sectoral collaboration, evidence-based interventions, and sustained investments in healthcare infrastructure and workforce capacity. By prioritizing maternal and child health resilience in pandemic preparedness and response efforts, India can mitigate the long-term consequences of the pandemic and ensure equitable access to quality health care.

## References

[1] Filip R, Gheorghita Puscaselu R, Anchidin-Norocel L, Dimian M, Savage WK (2022). Global challenges to public health care systems during the COVID-19 pandemic: a review of pandemic measures and problems. J Pers Med.

[2] Rosenthal A, Waitzberg R (2023). The challenges brought by the COVID-19 pandemic to health systems exposed pre-existing gaps. Health Policy Open.

[3] Alabi QK, Oyedeji AS, Kayode OO, Kajewole-Alabi DI (2023). Impact of COVID-19 pandemic on mother and child health in Sub-Saharan Africa: a review. Pediatr Res.

[4] Melaku T, Assefa D, Gashe F, Getachew M, Kabeta T, Mekonnen Z (2023). Impact of COVID-19 pandemic on the availability of maternal and child health products and childhood vaccines. J Pharm Policy Pract.

[5] Kingsley JP, Vijay PK, Kumaresan J, Sathiakumar N (2021). The changing aspects of motherhood in face of the COVID-19 pandemic in low- and middle-income countries. Matern Child Health J.

[6] Singh AK, Jain PK, Singh NP, Kumar S, Bajpai PK, Singh S, Jha M (2021). Impact of COVID-19 pandemic on maternal and child health services in Uttar Pradesh, India. J Family Med Prim Care.

[7] Kasthuri A (2018). Challenges to healthcare in India: the five A's. Indian J Community Med.

[8] (2024). Maternal mortality in India: 1997-2003 trends, causes and risk factors. https://www.cghr.org/wordpress/wp-content/uploads/RGI-CGHR-Maternal-Mortality-in-India-1997%E2%80%932003.pdf.

[9] (2024). Trends in maternal mortality 2000 to 2017: estimates by WHO, UNICEF, UNFPA, World Bank Group and the United Nations Population Division. Geneva, Switzerland. https://www.unfpa.org/sites/default/files/pub-pdf/Maternal_mortality_report.pdf.

[10] Lim SS, Dandona L, Hoisington JA, James SL, Hogan MC, Gakidou E (2010). India’s Janani Suraksha Yojana, a conditional cash transfer programme to increase births in health facilities: an impact evaluation. Lancet.

[11] (2024). Janani Shishu Suraksha Karyakaram (JSSK). https://nhm.gov.in/index4.php.

[12] (2024). Pradhan Mantri Surakshit Matritva Abhiyan. https://pmsma.mohfw.gov.in/about-scheme/.

[13] Chakraborty AK (2024). Universal Immunization Programme--retrospect and prospect. Indian J Public Health.

[14] Sahoo KC, Negi S, Patel K, Mishra BK, Palo SK, Pati S (2021). Challenges in maternal and child health services delivery and access during pandemics or public health disasters in low-and middle-income countries: a systematic review. Healthcare (Basel).

[15] Sharma S, Singh L, Yadav J, Gupta U, Singh KJ, Rao MV (2023). Impact of COVID-19 on utilization of maternal and child health services in India: health management information system data analysis. Clin Epidemiol Glob Health.

[16] Singh T, Kaur R, Kant S, Yadav K, Gupta S (2023). Voices from the community: maternal healthcare experiences during the COVID-19 pandemic. Cureus.

[17] Kotlar B, Gerson EM, Petrillo S, Langer A, Tiemeier H (2021). The impact of the COVID-19 pandemic on maternal and perinatal health: a scoping review. Reprod Health.

[18] Miller MA, Sentz JT (2024). Vaccine-preventable diseases. Disease and Mortality in Sub-Saharan Africa.

[19] Onchonga D, Alfatafta H, Ngetich E, Makunda W (2021). Health-seeking behaviour among pregnant women during the COVID-19 pandemic: a qualitative study. Heliyon.

[20] Aranda Z, Binde T, Tashman K (2022). Disruptions in maternal health service use during the COVID-19 pandemic in 2020: experiences from 37 health facilities in low-income and middle-income countries. BMJ Glob Health.

[21] Jaiswal J, LoSchiavo C, Perlman DC (2020). Disinformation, misinformation and inequality-driven mistrust in the time of COVID-19: lessons unlearned from AIDS denialism. AIDS Behav.

[22] Hopkins KL, Underwood T, Iddrisu I (2023). Community-based approaches to increase COVID-19 vaccine uptake and demand: lessons learned from four UNICEF-supported interventions. Vaccines (Basel).

[23] Haldane V, De Foo C, Abdalla SM (2021). Health systems resilience in managing the COVID-19 pandemic: lessons from 28 countries. Nat Med.

